# Bone disease and oromaxillofacial disorders: a cross- sectional study in a Tanzanian pediatric population

**DOI:** 10.1186/s13023-025-03563-0

**Published:** 2025-02-17

**Authors:** Elias Isaack Mashala, Lluís Brunet-Llobet, Anastasiya Lapitskaya, Sol Balsells-Mejía, Ombeni Mrina, Jaume Miranda-Rius

**Affiliations:** 1 Department of Orthopedic Surgery and Traumatology, Mount Meru Regional Referral Hospital, Arusha, Tanzania; 2 Doctoral programme in Medicine and Translational Research, Line of Odontostomatology, Faculty of Medicine and Health Sciences, Hospital Sant Joan de Déu, University of Barcelona, Barcelona, Spain; 3 Department of Odontostomatology, Faculty of Medicine and Health Sciences, University of Barcelona, Barcelona, Spain; 4 Department of Dentistry, Hospital Sant Joan de Déu, University of Barcelona, Barcelona, Spain; 5 Hospital Dentistry and Periodontal Medicine Research Group, Institut de Recerca Sant Joan de Déu (IRSJD), Barcelona, Spain; 6 Department of Research Promotion and Management, Statistical Support. Hospital Sant Joan de Déu (HSJD), Barcelona, Spain; 7 Shalom Dental Surgery, GEMSA Specialist Polyclinic-Hospitals, Arusha, Tanzania

**Keywords:** Oromaxillofacial disorders, Bone diseases, Skeletal deformities, Congenital condition, Rare diseases, Dental abnormalities, Malocclusion

## Abstract

**Background:**

Certain bone diseases of congenital origin are associated with dental alterations and with oromaxillofacial (OMF) disorders. The objective of this study was to evaluate and compare the OMF alterations presented by patients affected by bone pathology with respect to a healthy population from the same geographical environment.

**Material & methods:**

A cross-sectional study was carried out at Mount Meru Regional Referral Hospital and Kaloleni secondary school in Arusha, Tanzania. The patients with bone pathologies (*n* = 60) were consecutively recruited from the hospital, while the controls (*n* = 581) comprised a population of healthy students from the school, which was located in the same neighbourhood as the hospital. In the case group, the different bone pathologies were divided into two subgroups: (i) disorders in cellular metabolism (DCM); and (ii) disorders of bone growth/deformity (DGD). Musculoskeletal and oral clinical examinations were performed in both groups.

**Results:**

The case group presented significantly higher values of moderate and severe inflammation on the Löe & Silness Gingival Index (GI 2: 65%, GI 3: 25%) than the control group (*p* < 0.001), where mild inflammation predominated (GI 1: 88%). The case group also had higher scores for decayed, missing and filled teeth. Dental fluorosis was reported in 75.2% of controls and in only 26.6% of cases, the differences being clearly significant (*p* < 0.001). Significant differences for fluorosis were also reported between the two subgroups (*p* < 0.001), with a higher incidence for the DCM subgroup (43.8%). Twenty-two patients (36.7%) in the case group displayed clinical absence of teeth: the rate was significantly higher in the DGD subgroup (*n* = 15, 50%) than in the DCM subgroup (*n* = 8, 25%) (*p* = 0.045). In relation to the type of dental occlusion, the group with bone pathology presented a significant predominance of Angle class II - III malocclusions (*p* < 0.001). Craniofacial abnormalities were more frequent in the DGD subgroup, although the difference was not significant. The spine was normal in 41 patients (68.3%) and the differences between subgroups were not significant. Pathological fractures were significantly more frequent in the DGD subgroup (50% vs. 6.3%; *p* < 0.001). Assessing whether there was a relationship between malocclusion and skeletal deformities (spine and upper limb) in the case group, subjects with upper limb deformity (*n* = 16) presented significant differences for inverted overjet (*p* = 0.031).

**Conclusion:**

Patients with bone disease had worse oral health and more severe dental malocclusion than controls. The results presented here may help to raise awareness among orthopedic and pediatric professionals of abnormalities related to OMF conditions in childhood.

**Supplementary Information:**

The online version contains supplementary material available at 10.1186/s13023-025-03563-0.

## Background

It is well known that certain bone diseases of congenital origin are associated with dental alterations (for example, alterations in number, morphology and eruption processes) and with oromaxillofacial (OMF) disorders. Investigating the pathophysiology of these entities will help to understand their clinical expression. Although a priori these entities are rare and occur in only one in 200,000 people, the total number of patients affected is nonetheless considerable [1].

Rare diseases are difficult to diagnose and are easily confused. Those affecting the skeleton, known as skeletal dysplasias, are no exception [1]. A more thorough understanding of rare diseases and their clinical manifestations is likely to lead to significant diagnostic and therapeutic advances [2].

Skeletal dysplasias typically present with short stature in childhood, but due to their heterogeneity, their musculoskeletal effects range notably in terms of severity– from premature arthritis in average height individuals in the mildest cases to severe short stature and death in the perinatal period in the most profoundly affected. In the pediatric population, the patients most likely to be seen by a pediatrician or orthopedic surgeon are the ones that present with short stature. The spine and limbs are frequently affected. In addition to musculoskeletal alterations, deficits in hearing and vision and neurological, respiratory, cardiac, and renal disorders are frequently recorded, as are psychological problems [3].

However, disorders related to the OMF complex in skeletal dysplasia can often go unnoticed or may be described in a generic and superficial way. Certain studies have reported increased levels of OMF disorders in children and adolescents with rare genetic diseases affecting skeletal development, which have a clearly negative impact on quality of life [4–6].

The correct genetic formation of the skeleton and dentition requires the integration of numerous processes that begin in the early stages of embryological development. These processes include the modelling of the head, limbs and OMF structures through phenomena such as (i) cell migration and proliferation, (ii) differentiation into specialized cells, (iii) matrix secretion, (iv) biomineralization and (v) bone remodelling [1, 5]. Tooth development also depends on parallel processes involved in bone (skeletal) formation, although tooth remodelling processes do not involve physiological resorption.

Many clinical disorders of the bones and teeth during embryological development occur following disruption of any of the four stages of bone and tooth formation. These disruptions may be: (i) disorders of cell patterning and differentiation, for example fibrous dysplasia; (ii) disorders of growth with defective matrix function, for example osteogenesis imperfecta; (iii) disorders of metabolism and mineralization, for example hypophosphatemic rickets; and (iv) bone resorption (osteopetrosis) [1]. Miscellaneous bone diseases such as idiopathic scoliosis, neurofibromatosis, muscular dystrophy and arthrogryposis do not appear in the above classification of skeletal dysplasia but they are considered to have a high prevalence in the community [1].

The diagnosis of OMF disorders in children with bone disease is often overlooked, with the result that the disorders go untreated. Little information is available on the dental clinical manifestations in these patients or on the diagnostic approaches applied by pediatric orthopedic surgeons.

Some authors have investigated the possible relationship between bone disease and OMF alterations, but to our knowledge very few studies have been published in pediatric populations [1, 3, 6–8].

The majority of studies addressing the relationship between oromaxillofacial disorders and bone diseases focus on a single entity (e.g., osteogenesis imperfecta, mucopolysaccharidosis, etc.). Our bibliography search did not reveal any articles in which these bone pathologies were grouped together in relation to changes in the dental and/or cranial alterations.

The objectives of this study were to evaluate and compare the OMF alterations presented by patients with bone pathology with respect to a healthy population from the same geographical environment, and to analyse the clinical differences presented by the two subgroups of bone pathologies inside the case group.

## Materials and methods

A cross-sectional study following the STROBE checklist was carried out at Mount Meru Regional Referral Hospital and Kaloleni secondary school in the town of Arusha, northern Tanzania, between 2019 and 2022. The patients with bone pathologies were consecutively recruited from the hospital while the controls comprised a population of healthy students from the school, which was located in the same neighbourhood as the hospital. This study was part of a non-profit project approved in advance by the health authorities of Arusha City Council. Permission to conduct the study was obtained from the regional health authorities (ethical clearance certificate, Ref. no. CD / E.10/39/131), and the patients’ parents gave informed consent.

### Sample size

The calculation of the sample of patients with bone pathology (cases, *n* = 60) was made on the basis of the frequency of their visits to the Orthopedic outpatient service. Another factor considered was the patients’ capacity to undergo a clinical dental examination at the hospital. The control sample recruited was considerably larger (*n* = 581). This population had taken part in an earlier population-based study of dental fluorosis, carried out in the same area of influence. This sample size achieved a statistical power of 80% for the detection of effect sizes of 0.38 in two-sided t-tests, working with a significance level of 0.05 [8].

### Study area

The epidemiological characteristics of both groups were representative and homogeneous. The district of Kaloleni, with a population of approximately 11,650 inhabitants, has a low socioeconomic level, while the World Bank country classification defines the United Republic of Tanzania as a low-income economy. Arusha and its catchment area have a population of approximately two million inhabitants, with an average annual per capita income of US$499 [8].

### Inclusion and exclusion criteria

Patients aged between 6 and 18 years with bone disease, stable mental state and able to cooperate in dental or clinical assessment were included after their parents or caregivers had signed the consent forms and ethical clearance had been obtained. Patients with bone diseases related to trauma, infections and intellectual disability were excluded.

### Measurements

Convenience sampling was used to identify patients with bone pathology and to define them as the case group. Clinical examination of their musculoskeletal system was performed, and data on age, height, weight, skull, spine, and limbs were recorded. Radiological imaging (predominantly X-ray) of the affected part of the skeleton was performed. No additional imaging (for example, CT scan or MRI) was performed, nor any laboratory tests.

Because of the absence of molecular, genetic and chromosomal laboratory studies, none of the patients in the case group underwent tests to confirm the genetic diagnosis. Diagnosis was provisionally clinical and was based on thorough clinical reports and X-ray results recorded by two consultants (an orthopedic surgeon and a pediatrician).

Skeletal deformities such as alterations of upper or lower limbs were classified anatomically according to *the International Classification of Diseases*,* Ninth Revision (ICD9) codes 755.2 to 755.4 (for upper*,* lower and unspecified limb defects)* [9].

Limb deficiencies were defined as complete or partial limb absence. Cases with partial limb absence were subdivided further into three subgroups: (i) intercalary defects, defined as the absence or hypoplasia of a middle section of a long bone such as the femur or radius, with normal distal structures such as the hand, foot, or digits; (ii) terminal transverse defects causing the absence of all distal structures beyond a specific point perpendicular to the limb, such as absence of the lower half of the forearm and hand; and (iii) longitudinal defects, defined as the absence or hypoplasia of a bone parallel to the long axis of the limb and including preaxial, central, postaxial, and mixed pre- and postaxial longitudinal defects [9, 10]. Other deformities which were not applicable in this classification were also defined, such as hip and knee joint deformities, club hand and foot, and multiple complex deformities.

The oral clinical examination in both groups applied indices such as the Silness & Löe Plaque Index (PI) to assess oral hygiene [11]. The gums were assessed using the Löe & Silness Gingival Index (GI) [12]. The Decayed, Missing, Filled Teeth (DMF-T) index was used to determine the prevalence of caries and the need for dental treatment [13], and the Thylstrup-Fejerskov (TF) index to establish the degree of fluorosis [14]. Dental clinical abnormalities (dental agenesis, supernumerary teeth, shape and position of abnormalities) were recorded, and when possible a panoramic x-ray was requested. Dental absences were recorded when one or more teeth were not present in the mouth. As regards occlusion disorders, angle classes were recorded as either overjet, overbite or crossbite.

### Data interpretation and statistical analysis

The dental assessments were carried out consecutively by two trained examiners, EIM and OM, who performed clinical assessments of oral hygiene, dental caries, dental abnormalities (size, shape, and number), malocclusion, and a basic clinical examination of the temporal mandibular joint (TMJ). The measurement system was calibrated before the recording of the data, and the interrater agreement kappa index was 0.84. Descriptive statistics were analysed for both samples (cases and controls). In the case group, the different bone pathologies were divided into two subgroups considered to present a similar or related etiopathogenesis for their bone manifestation: (i) disorders in cellular metabolism (DCM), and (ii) disorders of bone growth/deformity (DGD), in order to identify any intragroup differences in their dental alterations.

A descriptive analysis was performed for all groups and subgroups studied. Frequency tables were generated for categorical variables and the corresponding descriptive statistics were calculated for numerical variables. Normality in the distribution of numerical variables was assessed with QQ plots.

For categorical variables, the chi-square test or Fisher’s exact test was used to study differences between cases and controls and between different bone pathologies, and the Student t-test or Mann-Whitney U-test (depending on the distribution of the variable) for numerical variables. *P*-values of 0.05 or less were considered significant. The analysis was performed using the Statistical Package for the Social Sciences (SPSS for Window, version 23.00 IBM Inc, Amonk, NY, USA).

## Results

The total sample comprised 641 subjects: 60 cases with bone disease and 581 healthy controls. The mean age was significantly lower in patients with bone pathology (10.57 years, SD 3.76) than in the healthy population (15.69 years, SD 1.45); (*p* < 0.001) but the gender distribution was similar in the two groups (Table 1).

**Table 1:** Sociodemographic data and oral manifestations

Variable:	Control group *(N = 581)*	Case group *(N = 60)*		*P*-value	*Case group (N = 60)*		
					*DCM (N = 32)*	*DGD (N = 28)*	*P*-value
***Age: (years)***	*15.69 (1.45)*	*10.57 (3.76)*		0.001* ^***(1)***^	*11.1 (3.6)*	*10.0 (3.9)*	*0.279* ^*(1)*^
***Sex: Female*** *** Male***	*283 (48.7%)* *298 (51.3%)*	*28 (46.7%)* *32 (53.3%)*		*0.868* ^*(3)*^	*12 (37.5%)* *20 (62.5%)*	*16 (57.1%)* *12 (42.9%)*	*0.128* ^*(3)*^
***Mean height: (cm); (SD)***	*-*	*-*			*130.4 (19.1)*	*117.5 (23.2)*	0.021* ^*(1)*^
***Mean weight: (kg)***	*-*	*-*			*39.1 (12.7)*	*30.5 (11.5)*	0.008* ^*(1)*^
***Dentition***:							
***Temporary*** ***Mixed*** ***Permanent***	*-* *-* *581 (100%)*	*10(16.7%)* *24(40.0%)* *26(43.3%)*			*4 (12.5%)* *11 (34.4%)* *17 (53.1%)*	*6 (21.4%)* *13 (46.4%)* *9 (32.1%)*	*0.250* ^*(3)*^
***Plaque Index***:							
***PI 0*** ***PI 1*** ***PI 2*** ***PI 3***	*68 (11.7%)* *355 (61.1%)* *148 (25.5%)* *10 (1.7%)*	*6 (10.0%)* *32 (53.3%)* *15 (25.0%)* *7 (11.7%)*		*0.076* ^*(2)*^	*1 (3.1%)* *17 (53.1%)* *10 (31.3%)* *4 (12.5%)*	*5 (17.9%)* *15 (53.6%)* *5 (17.9%)* *3 (10.7%)*	*0.111* ^*(2)*^
***Gingival index***:							
***GI 0*** ***GI 1*** ***GI 2*** ***GI 3***	*59 (10.2%)* *511 (88.0%)* *11 (1.8%)* *0*	*0* *6 (10.0%)* *39 (65.0%)* *15 (25.0%)*		0.001* ^*(2)*^	*0* *1 (3.1%)* *21 (65.6%)* *10 (31.3%)*	*0* *5 (17.9%)* *18 (64.2%)* *5 (17.9%)*	*0.069* ^*(2)*^
***DMF-T***:							
***0*** ***1–2*** ***> 2***	*317 (54.6%)* *180 (31.0%)* *84 (14.5%)*	*34 (56.7%)* *11 (18.3%)* *15 (25.0%)*		*0.489* ^*(2)*^	*22 (68.8%)* *3 (9.4%)* *7 (21.8%)*	*12 (42.8%)* *8 (28.6%)* *8 (28.6%)*	*0.166* ^*(2)*^
***Dental fluorosis***:							
***No*** ***Mild*** ***Moderate*** ***Severe***	*144 (24.8%)* *179 (30.8%)* *203 (34.9%)* *55 (9.5%)*	*44 (73.3%)* *5 (8.3%)* *8 (13.3%)* *3 (5.0%)*		0.001* ^*(2)*^	*0 (80.0%)* *5 (7.1%)* *6 (8.6%)* *3 (4.3%)*	*0 (96.2%)* *1 (1.3%)* *2 (2.5%)* *-*	0.001* ^*(4)*^

(*) p-value ≤ 0.05: significant. Numbers next to the p-values show which test was used: Student t-test (1), Mann-Whitney U-test (2), chi-square test (3) or Fisher’s exact test (4)

Among patients with bone pathology, the DCM subgroup (*n* = 32) presented a predominance of congenital limb deficiencies and neural tube defects (11 patients, 34.4%, and 10 patients, 31.3%, respectively), followed by four patients (12.5%) with hypophosphatemic rickets and three (9.4%) with craniosynostosis. Other conditions recorded were osteopetrosis, fibrous dysplasia, mucopolysaccharidosis and bone fluorosis, with one patient (3.1%) in each case. In the DGD subgroup (*n* = 28), 11 patients (39.3%) presented osteogenesis imperfecta. Among the miscellaneous bone deformities recorded, arthrogryposis was reported in nine patients (32.1%) and neurofibromatosis in three (10.7%); two patients each (7.1%) presented idiopathic scoliosis and dysplasia, and finally one patient (3.6%) presented muscular dystrophy (Table 2).

**Table 2:** Bone disease pathology

Bone disease group (case):	Differential diagnosis		Freq. (%)
***Disorders in cellular metabolism (DCM):***	*Fibrous dysplasia*		1 (3.1%)
	*Hypophosphatemic rickets*		*5 (15.6%)*
	*Craniosynostosis*		*3 (9.4%)*
	*Osteopetrosis*		*1 (3.1%)*
	*Mucopolysaccharidosis*		*1 (3.1%)*
	*Cong limb bone deficiencies*		*11 (34.4%)*
	*NTD - Spina bifida*		*10 (31.3%)*
	*Fluorosis*		*1 (3.1%)*
	***Total***		***32 (53.3%)***
***Disorders of bone growth/deformity (DGD):***	*Osteogenesis imperfecta*		*11 (39.3%)*
	*Idiopathic scoliosis*		*2 (7.1%)*
	*Neurofibromatosis*		*3 (10.7%)*
	*Arthrogryposis*		*9 (32.1%)*
	*Muscular dystrophy*		*1 (3.6%)*
	*Dysplasia*		*2 (7.1%)*
	***Total***		***28 (46.7%)***

As regards sociodemographic data, the mean age was 11.1 years (SD 3.6) in the DCM subgroup and 10 years (SD 3.9) in the DGD subgroup. Neither age nor sex presented significant differences between the two subgroups (*P*-values of 0.279 and 0.128 respectively). However, height and weight differed significantly, as members of the DCM subgroup were taller and heavier than those in the DGD subgroup (*p =* 0.021 and *p =* 0.008 respectively) (Table 1).

With regard to the clinical examination of the TMJ, the mean mouth opening was 40 mm (range 40–45 mm); only five subjects presented pathological mouth opening deviation and only one articular noises. Regarding the gingival index (GI), the case group presented significantly higher values of moderate inflammation (39 patients, GI 2: 65%) and severe inflammation (15 patients, GI 3: 25%) than the control group (*p* < 0.001), where the inflammation was mild in 511 children (GI 1: 88%). Values ≥ 2 on the plaque index were obtained in 36.7% of cases and in 27.2% of controls. Neither the differences between cases and controls (*p* = 0.070) nor between cases (*p* = 0.111) were significant. The comparison of the subgroups revealed severe inflammation in 10 DCM patients (GI 3: 31.3%) but in only five DGD patients (GI 3: 17.9%), although the difference was not significant (*p* = 0.069) (Table 1).

DMF-T scores were higher in the case group, since 15 cases (25%) presented a DMF-T > 2 compared to 84 controls (14.5%), although the overall differences were not significant (*p* = 0.489). However, comparison of the case subgroups found higher rates of DMF-T scores > 2 in the DGD subgroup (eight patients, 28.6%) compared with seven (21.8%) in the DCM subgroup. Once again, the difference was not significant (*p* = 0.166) (Table 1).

Dental fluorosis was reported in 437 controls (75.2%) and in only 16 cases (26.6%), the differences being clearly significant (*p* < 0.001). Significant differences for fluorosis were also reported between the two subgroups (*p* < 0.001), with a higher incidence for the DCM subgroup (43.8%) than in the DGD subgroup (7. 1%) regardless of severity (Table 1).

Since hardly any radiological variables were recorded, the term “clinical absence of teeth” is used here, including entities such as agenesis, unerupted, impacted, retained or inclusion teeth. Alterations in dental positions such as ectopias and transpositions were observed in 13 patients with bone disease (21.7%), shape abnormalities in nine (15%) and clinical supernumerary teeth in five (8.3%); the differences between the subgroups were not significant. However, 22 patients (36.7%) in the case group displayed clinical absence of teeth. The rate was significantly higher in the DGD subgroup (*n* = 15, 50%) than in the DCM subgroup (*n* = 8, 25%) (*p* = 0.045) (Table 3).

**Table 3:** Dental clinical abnormalities and occlusion

Variable:	Control group *(N = 581)*	*Case group (N = 60)*	*P*-value	*Case group (N = 60)*		
Dental clinical abnormalities:				DCM *(N = 32)*	DGD *(N = 28)*	*P*-value
***Supernumerary teeth***:		*5 (8.3%)*		*2 (6.3%)*	*3 (10.7%)*	*0.657* ^*(4)*^
***Shape abnormalities***:		*9 (15%)*		*5 (15.6%)*	*4 (14.3%)*	*1.000* ^*(4)*^
***Position abnormalities***:		*13 (21.7%)*		*9 (28.1%)*	*4 (14.3%)*	*0.226* ^*(4)*^
***Dental agenesis***^***¥***^:		*22 (36.7%)*		*8 (25.0%)*	*14 (50.0%)*	0.045* ^*(3)*^
***Occlusion***:						
***Class I*** ***Class II*** ***Class III***	*562 (96.7%)* *16 (2.8%)* *3 (0.5%)*	*41 (68.3%)* *16 (26.7%)* *3 (5.0%)*	0.001* ^***(2)***^	*19 (59.4%)* *10 (31.2%)* *3 (9.4%)*	*22 (78.6%)* *6 (21.4%)* *0*	*0.083* ^*(2)*^
***Overjet***:						
***Inverted (< 0 mm)*** ***Edge to edge (0–1 mm)*** ***Normal (2–3 mm)*** ***Increased (> 3 mm)***	*0* *288 (49.6%)* *267 (46.0%)* *26 (4.5%)*	*5 (8.3%)* *36 (60.0%)* *16 (26.7%)* *3 (5.0%)*	0.002* ^***(2)***^	*3 (9.4%)* *16 (50.0%)* *9 (28.1%)* *4 (12.5%)*	*2 (7.1%)* *20 (71.4%)* *5 (17.9%)* *1 (3.6%)*	*0.703* ^*(2)*^
***Overbite***:						
***Open bite (< 0 mm)*** ***Edge to edge (0–1 mm)*** ***Normal (2–3 mm)*** ***Increased (> 3 mm)***	*0* *307 (52.8%)* *270 (46.5%)* *4 (0.7%)*	*3 (5.0%)* *36 (60.0%)* *17 (28.3%)* *4 (6.7%)*	*0.081* ^*(2)*^	*3 (9.4%)* *15 (46.8%)* *11 (34.4%)* *3 (9.4%)*	*0* *21 (75.0%)* *6 (21.4%)* *1 (3.6%)*	*0.239* ^*(2)*^
***Crossbite***:						
***No*** ***Yes***	*563 (96.9%)* *18 (3.1%)*	*46 (76.7%)* *14 (23.3%)*	0.001* ^***(3)***^	*29 (90.6%)* *3 (9.4%)*	*28 (100%)* *0 (0.0%)*	*0.241* ^*(4)*^

(*) p-value ≤ 0.05: significant. Numbers next to the p-values show which test was used: Student t-test (1),* Mann-Whitney U-test (2)*,* chi-square test (3) or Fisher’s exact test (4). (*^*¥*^*): clinical absence of teeth in the mouth*

In relation to the type of dental occlusion, Angle class I was recorded in 41 cases (68.3%) and in 562 controls (96.3%). Thus, the group with bone pathology presented a significant predominance of Angle class II - III malocclusions (*p* < 0.001). Despite the lack of significant differences in malocclusion between the two subgroups, three patients (9.4%) from the DCM subgroup presented Angle class III but none from the DGD subgroup. In relation to other dental occlusion values, the cases presented a significant trend towards an edge-to-edge and/or inverted overjet (41 cases, 68.3%, vs. 288 controls, 49.6%; *p* = 0.002), as well as posterior crossbite occlusion (14 cases, 23.3%, versus 18 controls, 3.1% *p* < 0.001); no such trend appeared for overbite (*p* = 0.081) (Table 3).

Global skull deformities such as plagiocephaly, brachycephaly and scaphocephaly were recorded in eight patients (13.3%) among the case group. Craniofacial abnormalities were more frequent in the DGD subgroup, although no significant differences were recorded (facial abnormalities; *p* = 0.130 and, cranial flattening; *p* = 0.404, craniosynostosis; *p* = 0.675). The spine was normal in 41 patients (68.3%) and the differences between subgroups were not significant (seven patients in the DCM subgroup, 21.9%, versus 12 in the DGD subgroup, 42.9% *p* = 0.081). However, more spinal deformities were found in the DGD subgroup (12 patients, 42.9% vs. 7 patients, 21.9%). Pathological fractures were significantly more frequent in the DGD subgroup (14 patients, 50%, vs. two patients, 6.3%) (*p* < 0.001) (Table 4).

**Table 4:** Skeletal manifestation

Variable: skeletal deformities	*Case group (N = 60)*		
	*DCM (N = 32)*	*DGD (N = 28)*	*P*-value
***Skull deformities***	*5 (15.6%)*	*3 (10.7%)*	*0.712* ^*(4)*^
***Facial shape deformities***	*2 (6.3%)*	*6 (21.4%)*	*0.130* ^*(4)*^
***Cranial flattening***	*2 (6.3%)*	*4 (14.3%)*	*0.404* ^*(4)*^
***Early craniosynostosis***	*4 (12.5%)*	*2 (7.1%)*	*0.675* ^*(4)*^
***Spine deformities***	*7 (21.9%)*	*12 (42.9%)*	*0.081* ^*(3)*^
***Upper limb deformities***	*8 (25.0%)*	*6 (21.4%)*	*0.391* ^*(3)*^
***Lower limb deformities***	*26 (81.3%)*	*25 (89.3%)*	*0.482* ^*(3)*^
***Pathological fractures***	*2 (6.3%)*	*14 (50.0%)*	< 0.001* ^*(4)*^

(*) p-value ≤ 0.05: significant. Numbers next to the p-values show which test was used: chi-square test (3) or Fisher’s exact test (4)

Upper limb deformities were recorded in 16 cases (26.7%). Congenital absence of upper limbs (phocomelia) and club hand were only described in two patients, both in the DCM subgroup. Four patients (50%) presented terminal transverse defects, most of them (three patients, 37.5%) below the elbow. Six patients in the DGD subgroup presented longitudinal defects, in four cases (66.7%) in the arm and forearm. No such defects were recorded in the DCM group (Tables 4 and 5).

**Table 5:** Upper and lower limb deformities

Anatomical classification^#^	Specific anatomical location	Case group	
		DCM (N = 32)	DGD (N = 28)
***Upper limb deformities***			
***Intercalary***	*Forearm*	*-*	*2 (33.3%)*
***Longitudinal***	*Arm and forearm*	*-*	*4(66.7%)*
***Terminal transverse***	*Below elbow*	*3(37.5%)*	*-*
	*MCP (fingers)*	*1(12.5%)*	*-*
***Congenital absence***	*Congenital absence*	*2(25.0%)*	*-*
***Club hand***	*Club hand*	*2(25.0%)*	*-*
***Multiple deformities***	*Multiple deformities*	*-*	*-*
**TOTAL**		**8 (25.0%)**	**6 (21.4%)**
**Lower limb deformities**			
***Intercalary***	*Femur/thigh*	*2 (7.7%)*	*-*
***Longitudinal***	*Tibia/fibula*	*1 (3.8%)*	*6 (24.0%)*
***Terminal transverse***	*Above knee (upper leg)*	**-**	*3(12.0%)*
	*Below knee (lower leg)*	*9(34.6%)*	*4(16.0%)*
	*MTP (toes)*	*2 (7.7%)*	*0(0.0%)*
***Multiple deformities***	*Multiple deformities*	*4(15.4%)*	*5(20.0%)*
***Congenital absence***	*Congenital absence*	*2(7.7%)*	**-**
***Miscellaneous bone deformity***			
***Hip***	*Hip dysplasia*	*-*	*2(8.0%)*
***Knee***	*Knee joint deformity*	*4(15.4%)*	*3(12.0%)*
***Foot***	*Club foot*	*2(7.7%)*	*2 (8.0%0)*
**TOTAL**		**26(81.3%)**	**25(89.3%)**

***#****Anatomical classification used by the International Classification of Diseases*,* Ninth Revision (ICD9) codes 755.2 to 755.4*

*(For upper*,* lower and unspecified limb defects). Two cases in the DCM had non-specific upper limb deformities*

Lower limb deformities were recorded in 51 cases (85%) in the bone pathology group. In this series terminal transverse defects were common in both subgroups, with defects either above or below the knee in the DCM subgroup; intercalary defects of the femur were less common, occurring in only two patients (7.7%) in this subgroup. Although rates of multiple deformities were similar in the two subgroups, congenital absence was recorded in only two patients (7.7%) in the DCM subgroup. Miscellaneous bone deformities were recorded in both subgroups, with a high prevalence of knee joint deformities. Despite the similar prevalence of club foot in the two subgroups (7.7-8%), no patients in the DCM subgroup presented hip dysplasia (Table 5).

We assessed whether there was a relationship between malocclusion and skeletal deformities (spine and upper limb) in the group of cases. No significant differences were found for spine deformity, but in subjects with upper limb deformity (*n* = 16), significant differences were found for overjet (inverted) (*n* = 4; 25.0% vs. *n* = 1; 2.3%, *p* = 0.031) (Table 6).

**Table 6:** Relationship between malocclusion and skeletal deformities

Variable	Spine deformity			Upper limb deformity		
	No (*N*=41)	Yes (*N*=19)	*P*-value	No (*N*=44)	Yes (*n*=16)	*P*-value
***Malocclusion***						
***Class I*** ***Class II*** ***Class III***	28(68.3%) 10(24.4%) 3(7.3%)	13(66.7%) 6 (33.3%) -	0.861 ^*(2)*^	32(72.7%) 11(25.0%) 1(2.3%)	9(56.3%) 5(31.2%) 2(12.5%)	0.338 ^*(2)*^
***Overjet***						
***Inverted (<0 mm)*** ***Edge to edge (0–1 mm)*** ***Normal (2–3 mm)*** ***Increased (>3 mm)***	3(7.3%) 24(58.5%) 10(24.4%) 4(9.8%)	2(10.5%) 12(63.2%) 4(21.1%) 1(5.3%)	0.539 ^*(2)*^	1(2.3%) 27(61.4%) 12(27.3%) 4(9.0%)	4(25%) 9(56.3%) 2(12.5%) 1(6.2%)	0.031* ^*(2)*^
***Overbite***						
***Open bite (<0 mm)*** ***Edge to edge (0–1 mm)*** ***Normal (2–3 mm)*** ***Increased (>3 mm)***	3(7.3%) 23(56.1%) 12(29.3%) 3(7.3%)	0 13(68.4%) 5(26.3%) 1(5.3%)	0.856 ^*(2)*^	1(2.3%) 28(63.6%) 12(27.3%) 3(6.8%)	2(12.5%) 8(50.0%) 5(31.3%) 1(6.2%)	0.646 ^*(2)*^
***Open bite***	3(7.3%)	-	0.545 ^*(4)*^	1(2.3%)	2(12.5%)	0.171 ^*(4)*^
***Crossbite***	8(19.5%)	6(31.6%)	0.304 ^*(3)*^	9(20.5%)	5(31.3%)	0.382 ^*(3)*^

(*) p-value ≤ 0.05: significant. Numbers next to the p-values show which test was used: Student t-test (1), Mann-Whitney U-test (2), chi-square test (3) or Fisher’s exact test (4)

Finally, the complementary diagnostic methods performed in the case group comprised radiological explorations recorded according to anatomical region: X-rays of the limbs were performed in 30 patients in the case group (50%), of the spine in 19 (31.7%) and of the skull in six (10%). None of the tests showed significant differences between the two subgroups. Clinical assessment and X-rays were the methods most commonly used to diagnose bone and tooth disease. Panoramic X-ray was conducted in only eight patients (13.3%), and none underwent laboratory tests to confirm bone disease (Table 7).

**Table 7:** Diagnostic test

Variable:	*Case group (N = 60)*		
	*DCM (N = 32)*	*DGD (N = 28)*	*P*-value
***Diagn. method: Skull X-Rays***	*2 (6.3%)*	*4 (14.3%)*	*0.404* ^*(4)*^
***Diagn. method: Spine X-Rays***	*7 (21.9%)*	*12 (42.9%)*	*0.081* ^*(3)*^
***Diagn. method: Limbs X-Rays***	*14 (43.8%)*	*16 (57.1%)*	*0.301* ^*(3)*^
***Diagn. method: Panoramic X-Rays***	*5 (15.6%)*	*3 (10.7%)*	*0.712* ^*(4)*^

(*) p-value ≤ 0.05: significant. Numbers next to the p-values show which test was used: chi-square test (3) or Fisher’s exact test (4)

## Discussion

The WHO defines rare diseases as those that affect 65 per 100,000 people [15]; they are usually chronic, degenerative, progressive and incapacitating and they often entail a loss of quality of life [16]. Seventy-five per cent of rare diseases affect children and emerge for the first time during childhood; 72% are of genetic origin [17].

Rare diseases are characterized by a wide diversity of symptoms and signs that vary not only from disease to disease but also among patients suffering from the same condition. Due to the low prevalence of each disease, medical expertise and knowledge are scarce, the research carried out is limited, and the care available is inadequate. Especially in developing countries patients with rare disease may be neglected by health systems, and are often denied diagnosis, treatment, and the benefits of research [17]. Relatively common symptoms may hide underlying rare diseases, which leads to misdiagnosis and delays in treatment. The frequent absence of any effective cures adds to the suffering endured by patients and their families [17].

Certain rare bone diseases of congenital origin are associated with OMF disorders. To explain the connection, we must go back to the early stages of embryological development and consider the trilaminar germinative disc. The teeth derive from two of the primary germ layers, the ectoderm and the mesoderm. The development of the neural crest plays a key role in odontogenesis; around the sixth week there is a cellular contribution from the neural crest which will give rise to ectomesenchymal lamella. The ectomesenchyme is the origin of dentition and it will provide material for dentine and pulp. From this the cementum and periodontal ligament will also derive, including the alveolar bone. The trilaminar disc includes (i) the ectoderm, which will generate the nervous system, the sense organs, the epidermis and its appendages such as hair, nails, glands, and enamel; (ii) the mesoderm, which will give rise to the musculature, much of the connective tissue, cartilage and bone, the circulatory system, blood, and the urogenital system; and (iii) the endoderm, which is related to the primitive gut, from which much of the digestive tract, the adnexal glands and the respiratory tract all derive [18]. As a result, a better understanding of the pathophysiology of certain bone diseases associated with OMF and dentition disorders would expand our knowledge of their clinical expression [1].

Our study describes alterations in dentition and OMF disorders and the possible relationships in children with bone pathology, in comparison to a control population from the same area recruited at a publicly-funded school in Arusha, northern Tanzania. We used medical records to establish the clinical diagnosis of bone pathology, carrying out careful physical examinations and on occasion consulting radiological reports [3]. We stress that in most East African countries, diagnoses of genetic diseases associated with skeletal deformities are only presumptive: the relative lack of health resources makes it very difficult to carry out genetic testing and specific DNA analyses to confirm diagnoses. In this geographical setting, diagnoses of most genetically skeletal deformities are only provisional [3].

We found that patients with bone pathology were significantly younger than the healthy population. This finding corroborates those of other studies where a high proportion of patients with oral problems were preschoolers, especially in areas with lower socioeconomic levels and limited resources [19, 20]. All our children attended a publicly-funded school and were from families with low socio-economic status.

With regard to our bone pathology subgroups, in the DCM group congenital limb deficiency was the most frequent, followed by neural tube defects, while in the DGD subgroup, osteogenesis imperfecta was the most prevalent, followed by arthrogryposis. Among cases as a whole, congenital limb deficiency, neural tube defects and osteogenesis imperfecta were the most prevalent diseases. Although these diseases are reported as “rare” in the general population, their true incidence in an East African/Tanzanian medical-social environment is likely to be higher due to under-diagnosis [3].

Patients in the DCM subgroup were taller and heavier than their DGD peers. Although height and weight were not recorded in the control group for comparison, our patients in the case group were generally shorter and thinner than average (according to the <-2Z-score) with respect to the WHO growth charts for the 5–19 year age group [21]. These findings are in agreement with the typical association of severely short stature in children with skeletal dysplasia [3].

In our case group, the mean maximum mouth opening did not differ between males and females, in line with previous reports [22, 23]. Rates of pathological mouth deviation, temporomandibular joint disorder (TMJ) and articular noises were low in both subgroups, with no significant differences between them.

Oral health was measured by the GI, PI and DMF-T indices. Multicenter studies have reported that individuals with poor oral hygiene (with or without a rare disease) are more likely to present dental caries [20]. Patients with bone pathology presented significantly higher values of moderate to severe gingivitis. Similar findings were reported by other authors in patients with mucopolysaccharidosis, and osteogenesis imperfecta [24].

Parallel to bone pathology, factors such as dietary choices, socio-demographic variables, access to services and life course may also contribute to poor oral health [4, 20, 25]. The prevalence of tooth decay was higher in the case group (DMF-T > 2), but the difference was not significant. Clear associations have been reported between dental caries, skeletal diseases and oral hygiene [26]. Other factors involved include the lack of well-trained and skilled professionals, the restricted availability of dental services and their high cost, and the limited information available to parents regarding the importance of oral health. A direct link has been reported between access to oral health care services and the absence of rare genetic disease [25, 26].

Our study showed a significantly higher prevalence of dental fluorosis in controls than in cases, reflecting their older age and their higher rates of permanent dentition. Fluorosis was also significantly more common in the DCM subgroup than in the DGD subgroup, independently of severity. In the Arusha region the fluoride content of the drinking water is approximately 3.6 mg/l, well above the level recommended by the WHO (0.5-1 mg/l) [27]. The high use of magadi as a food additive is significantly associated with severe dental fluorosis (TF grade 5–9) [22, 28]. Excess fluoride leads to a defective formation of the enamel, thus slowing the maturation of the tissue in permanent teeth during the critical period of the first six years of life. A high concentration of fluoride has also been shown to increase the severity of dental caries [29–31].

Jackson et al. reported that during the period of enamel formation fluoride concentrations of 1ppm in drinking water, or even lower, may cause mild fluorosis. However, infants who drink water with fluoride levels of 1 ppm present protection against decay compared to those who drink water practically without fluoride. The same authors concluded that although mild fluorosis may have slight aesthetic effects, the protective role of fluoride against caries should be the primary consideration [8, 32].

One drawback of the present study was the fact that some of the variables obtained in the cases could not be compared with the controls, as they were not studied during data collection. Examples are dental anomalies (shape, position, number), TMJ dysfunction and articular deviation which were investigated only in the case group, and so only intragroup comparison is possible. We replaced the term “agenesis”, which requires radiological confirmation, with the term clinical absence of teeth (tooth in the arch - not erupted). There was a high prevalence of clinical tooth absences in the bone disease group (25%), which was significantly more common in the DGD subgroup than in the DCM subgroup, in agreement with other authors [2]. The case group presented a prevalence of clinical supernumerary teeth of 8.3%, although this rate is probably an underestimation because the majority were not confirmed by dental X-rays; in any case it is much higher than in healthy populations, in which the rates of supernumerary teeth and dental agenesis are 4.5% and 3.8% respectively [33].

We found malocclusion to be more frequent in the cases than in the controls. There was a clearly significant association between malocclusion Angle Class II and III in the bone pathology group; similar results have been published elsewhere [25, 26]. Our results for edge-to-edge bite and/or inverted overjet were also in line with other reports related to osteogenesis imperfecta, fibrous dysplasia, and hypophosphatemic rickets [34]. We have not found any previous studies of the dental relationship of the maxillary and mandibular arches in this country. One study in a Nigerian population reported a predominance of Angle class II and III malocclusions compared with type I, but another study in the same setting found Angle class I occlusion to be the most frequent [35]. Other studies examining the differences between ethnic groups with regard to craniofacial measurements and angles have helped to describe facial morphology [36].

Skeletal deformities were most commonly found in the upper and lower limbs and the spine. In the lower limbs, more defects were seen in the DCM group, although the difference was not significant. Among miscellaneous bone deformities, the knee joint was a frequent site. Overall, no significant differences were observed between the two subgroups. Collaborative studies have reported similar skeletal manifestations of genetic disorders, but focused on developmental or metabolic disorders or on miscellaneous entities [1, 37–39]. In our study we went beyond previous reports, in that we combined some of the etiologically-related syndromes or diseases to form subgroups, and then compared the differences.

The DGD subgroup presented more spinal deformities than the DCM group, although the difference was not significant. Disorders manifesting with spine deformity included kyphoscoliosis, osteogenesis imperfecta, neurofibromatosis, arthrogryposis, muscular dystrophy and dysplasia. Previously, neuromuscular scoliosis, predominantly of the thoracolumbar curve, has been reported in most patients with arthrogryposis [40]. Kyphosis and other vertebral deformities have often been seen in patients with osteogenesis imperfecta, and a high prevalence of spine deformity has been observed in osteopetrosis [41] and in neurofibromatosis [42].

Among cases, pathological fractures were significantly more prevalent in the DGD subgroup than in the DCM group. In DGD patients, growth disorders, especially defective matrix function, contribute to bone fragility and low density. In this subgroup most patients had osteogenesis imperfecta.

The evidence of the clinical and genetic heterogeneity of skeletal disorders has led to the creation of various classifications of these disorders based on their clinical and radiological features and, subsequently, their molecular and embryological features [18]. Genetic analysis is highly recommended, but in East Africa patients suffering from bone diseases do not have access to the sophisticated metabolic tests that might allow diagnosis.

The diseases of the skeletal system studied here share a range of rare clinical pictures, and as a result their diagnosis may take years. During this time, caregivers are faced with problems related to the shortage of specialized professionals able to make accurate diagnoses and also the unavailability of the complementary tests necessary to speed up the process, such as genetic and laboratory tests [43, 44]. As a consequence, the pediatric population with rare bone diseases is often managed by specialized teams (neurology, cardiology, psychiatry, orthopedics, physiotherapy, ophthalmology, otolaryngology), while dental care takes a back seat. This, in turn, aggravates existing dental problems and reduces the overall quality of life in patients with diseases of the skeletal system [45]. The limited access to dental care in this population impacts their oral health and reduces the likelihood of early intervention, with the result that the treatments eventually indicated tend to be more radical and more costly. The physical restrictions that this group endures may also impair their ability to perform adequate oral care [46].

### General limitations and future recommendations

The precarious economic situation of the parents or guardians of these children made it difficult to collect more radiological data. For this reason, one limitation of this study was the negligible number of participants who provide dental X-rays. Indeed, with regard to anomalies such as absence of teeth, most participants underwent only a clinical examination and so the recording of this deficiency was incomplete. Ideally, all patients with clinical absence of teeth would undergo an orthopantomography to provide radiological confirmation of the diagnosis. In East Africa as a whole, bone and oromaxillofacial diseases are treated by pediatricians and orthopedic surgeons, since they are the first practitioners to see these patients. In Tanzania, given the severe shortage of dentists. it tends to be these medical doctors who detect the possible oromaxillofacial alterations associated with bone diseases. In developing countries, the possibility of obtaining a definitive diagnosis (genetic and/or molecular) in the case of uncommon diseases is practically non-existent, and health professionals are rarely able to provide sufficient information.

This study may help to draw attention in the field of health sciences to certain rare or minority diseases in East Africa. The creation of guidelines for general practitioners, pediatricians, dentists and nurses in Tanzania will improve their ability to detect clinical signs compatible with certain bone diseases and oromaxillofacial disorders. The patients affected are particularly vulnerable from a psychological, social, economic and cultural point of view, and so studies in this area are likely to have a positive impact on their quality of life.

## Conclusion

Our findings show that patients with bone disease had worse oral health and more severe dental malocclusion than controls, especially with regard to caries, gingivitis and Angle Class II-III. Dental agenesis was more prevalent in the DGD subgroup, but dental fluorosis predominated in DCM.

The results presented here may help to raise awareness among orthopedic and pediatric professionals regarding abnormalities related to OMF conditions in childhood. These findings may also contribute to the development of guidelines for the clinical and radiological evaluation and diagnosis of these disorders in areas with limited resources.

## Electronic supplementary material

Below is the link to the electronic supplementary material.

Supplementary Material 1

## Data Availability

The datasets used and/or analysed during the current study are available from the corresponding author on request.

## Abbreviations

OMF: Oromaxillofacial
PI: Silness & Löe Plaque Index
GI: Löe & Silness Gingival Index
DMF-T: Decayed, Missing, Filled Teeth index
DCM: Disorders in cellular metabolism
DGD: Disorders of bone growth/deformity
