# Supplementary material for: Bone disease and oromaxillofacial disorders: a cross- sectional study in a Tanzanian pediatric population
Source: Orphanet J Rare Dis. 2025 Feb 17;20:77. doi: 10.1186/s13023-025-03563-0 (PMC11834300; doi:10.1186/s13023-025-03563-0)
Supplement: Supplementary file 1 — Supplementary Material 1 [file 13023_2025_3563_MOESM1_ESM.doc]

Dear Editor of Orphanet Journal of Rare Diseases,

I am writing to inform you that the first author of this manuscript*, Elias Isaack Mashala, is a traumatologist at the Mount Meru Hospital of Arusha, Tanzania, East Africa. Dr. Mashala is currently enrolled in a Doctoral programme at the University of Barcelona.

In view of his limited financial resources, as a PhD student, and the fact that Tanzania is a developing country, I would be grateful if you could consider granting us a waiver of exemption to avoid publication fees.

***Title:** *Bone diseases and oromaxillofacial disorders: cross sectional study in a Tanzanian pediatric population*.

Yours sincerely,

Corresponding author

Jaume Miranda-Rius, MD, DDS, PhD
